# *Drosophila* Adiponectin Receptor in Insulin Producing Cells Regulates Glucose and Lipid Metabolism by Controlling Insulin Secretion

**DOI:** 10.1371/journal.pone.0068641

**Published:** 2013-07-12

**Authors:** Su-Jin Kwak, Seung-Hyun Hong, Rijan Bajracharya, Se-Yeol Yang, Kyu-Sun Lee, Kweon Yu

**Affiliations:** 1 Neurophysiology Research Group, Bionano Centre, Korea Research Institute of Bioscience and Biotechnology (KRIBB), Daejeon, Korea; 2 Department of Functional Genomics, University of Science and Technology (UST), Daejeon, Korea; John Hopkins University School of Medicine, United States of America

## Abstract

Adipokines secreted from adipose tissue are key regulators of metabolism in animals. Adiponectin, one of the adipokines, modulates pancreatic beta cell function to maintain energy homeostasis. Recently, significant conservation between *Drosophila melanogaster* and mammalian metabolism has been discovered. *Drosophila* insulin like peptides (Dilps) regulate energy metabolism similarly to mammalian insulin. However, in *Drosophila*, the regulatory mechanism of insulin producing cells (IPCs) by adipokine signaling is largely unknown. Here, we describe the discovery of the *Drosophila* adiponectin receptor and its function in IPCs. *Drosophila* adiponectin receptor (dAdipoR) has high homology with the human adiponectin receptor 1. The dAdipoR antibody staining revealed that dAdipoR was expressed in IPCs of larval and adult brains. IPC- specific *dAdipoR* inhibition (*Dilp2>dAdipoR-Ri*) showed the increased sugar level in the hemolymph and the elevated triglyceride level in whole body. *Dilps* mRNA levels in the *Dilp2>dAdipoR-Ri* flies were similar with those of controls. However, in the *Dilp2>dAdipoR-Ri* flies, Dilp2 protein was accumulated in IPCs, the level of circulating Dilp2 was decreased, and insulin signaling was reduced in the fat body. In *ex vivo* fly brain culture with the human adiponectin, Dilp2 was secreted from IPCs. These results indicate that adiponectin receptor in insulin producing cells regulates insulin secretion and controls glucose and lipid metabolism in *Drosophila melanogaster*. This study demonstrates a new adipokine signaling in *Drosophila* and provides insights for the mammalian adiponectin receptor function in pancreatic beta cells, which could be useful for therapeutic application.

## Introduction

Mammalian adipokines are produced and secreted from adipose tissue. They play a key role in maintaining energy homeostasis through inter-organ communications. Adiponectin, one of the adipokines, has multiple beneficial roles for regulating energy homeostasis, inflammation, and apoptosis [1], [2]. Two adiponectin receptors, AdipoR1 and AdipoR2, are seven transmembrane domain proteins with inverted topology compared to G-protein coupled receptors [3]. AdipoR1 has a higher binding affinity to the globular form of adiponectin whereas AdipoR2 has a higher binding affinity to the full length adiponectin [3]. *AdipoR1* and *2* double knockout mice increase the triglyceride level in the liver and exhibit insulin resistance and glucose intolerance, demonstrating that AdipoR1 and 2 regulate lipid and glucose homeostasis [2], [4]. In the skeletal muscle and liver, adiponectin receptors activate AMPK (AMP-activated protein kinase), PPAR-alpha, and p38 MAPK to increase the insulin sensitivity [3]. An adaptor protein APPL1 binds to adiponectin receptors, which activates AMPK and p38 MAPK in the skeletal muscle [5]. However, the mechanism of how adiponectin receptors activate downstream effectors is not made clear and the adiponectin receptor signaling identified in the skeletal muscle is not always applicable in other tissues. A recent study showed that adiponectin receptors are associated with ceramidase activity and regulate cell apoptosis by adjusting the balance between ceramide and sphingosine-1 phosphate levels [6]. Although *AdipoR1* and *2* are expressed in pancreatic beta cells [7], [8], the function of adiponectin and AdipoRs in IPCs is less studied than in insulin target tissues such as liver and skeletal muscle [1], [2]. *Adiponectin* knockout mice show impaired insulin secretion and intravenous injection of adiponectin to C57BL/6 mice induces insulin secretion [9], [10]. These studies indicate that adiponectin regulates insulin secretion but IPC-specific modulation of *AdipoR* in the animal model has not been demonstrated to show that adiponectin directly regulates insulin secretion through AdipoR.

During the last decade, significant conservation and parallelism were discovered between *Drosophila* and the mammalian metabolism. For example, *Drosophila insulin like peptides* (*Dilps*) regulate growth, energy metabolism, stress response, aging, and reproduction functions similar to that of mammalian Insulin/IGF signaling. Ablation of IPCs or deletion of *Dilp* genes results in decreased body size, retarded growth, and diabetic phenotypes such as an elevated circulating sugar level and altered stored lipid and carbohydrate levels [11]–[15]. There are eight *Dilp* genes in *Drosophila* genome, and four of them (*Dilp* 1, 2, 3 and 5) are expressed in IPCs of the brain. Recent studies demonstrate that Dilp production in IPCs is regulated by multiple factors such as neuropeptides, neurotransmitters, microRNA, O-GlcNAc metabolism [16]–[23]. However, Dilp secretion is not well studied in the *Drosophila* IPCs. Recently, mammalian leptin like *unpaired 2* (*upd2*) signaling was discovered in *Drosophila*. When sugar and lipid are fed, Upd2 protein is produced from the fat body and regulates Dilp secretion through GABAnergic neurons in the fly brain [24]. In this report, we present the identification of *Drosophila* adiponectin receptor and its function on insulin secretion in IPCs of the fly brain.

## Materials and Methods

### *Drosophila* Culture and Stocks

*Drosophila melanogaster* were cultured at 25°C on standard cornmeal, yeast, sugar, agar diet. The stocks used in this study were *UAS-dAdipoR-RNAi* (VDRC 40936), *w-* and *UAS-AUG-DsRed* (Bloomington Stock Center), *UAS-secGFP* (M. González-Gaitán, University of Geneva), *Dilp2–Gal4* (E. Rulifson, University of California, San Francisco), *UAS-Dilp^FLAG^* [25] (P. Leopold, Institut Valrose Biologie), and *UAS–Dilp2* [16] (G. H. Lee, University of Tennessee). Unless otherwise indicated, 3–5 day old adult flies or third instar feeding larvae were used in experiments.

### Identification of *Drosophila* Adiponectin Receptor Sequence

To find *Drosophila* orthologs of human adipokine and adipokine receptors, NCBI standard protein blast program blastp was used (http://blast.ncbi.nlm.nih.gov). Non-redundant protein sequence database of *Drosophila melanogaster* was blasted with the human adipokine and adipokine receptors protein sequences.

### Measurement of *Drosophila* Body Weight, Size and Wing Size

To synchronize larval growth, the eggs were collected on the grape juice plate for 2 h and after 24 h, 50 hatched 1st instar larvae were transferred to a fly food vial. At 106–108 h after egg laying, the larval weight was measured. Then, the larvae were boiled for 3 minutes to measure the body length. For adult fly weight and wing length, 5 day-old male flies were used. More than 30 flies were used for each measurement.

### Measurement of Total Body Triglyceride Level

In each time, 10 larvae or adult flies were ground in PBS solution and centrifuged. The supernatant was used for the analysis. Total glycerol and triglyceride levels were measured using a serum triglyceride determination kit (Sigma). The protein levels were measured in the same samples to normalize the triglyceride level.

### Measurement of Trehalose and Glucose Levels in the Hemolymph

Larvae were starved on the water soaked filter paper for 4 h and 7.5% yeast/7.5% sucrose solution was fed for 30 mins. Adult flies were starved on 0.8% PBS-agar for 24 h and refed normal fly food for 2 h. Hemolymphs were collected from ten to fifteen flies. The concentrations of trehalose and glucose were measured as previously described [26].

### Dilp2-FLAG ELISA Assay

Dilp2-FLAG ELISA assay was performed as previously described [27] with some modifications. 0.5 µl of hemolymph was collected from feeding third-instar larvae and diluted in PBS. The wells of the Immuno 96-well plate (Maxisorp™, Nunc International) were coated overnight at 4°C with the diluted hemolymph. The next day, the wells were cleared and processed for ELISA assay. The primary antibody (anti-FLAG M2 antibody, 1∶500, Sigma) was added to the wells and incubated at RT for 2 h. The HRP-conjugated secondary antibody (1∶1000, SantaCruz Biotechnology) was treated at RT for 1 h. TMB solution (Thermo Scientific; Rochester, NY) was used for color development, and the optical density was measured at 450 nm. The standard curve for quantification was generated with serially diluted 3×FLAG peptide (Sigma).

### Starvation and High Fat Diet Resistance Assays

The starvation assay was performed as in Broughton et al., 2008. The high fat diet food was made by adding 20% coconut oil (vol/vol) to the normal fly food [28]. 3–5 day old female flies were collected from the normal fly food and transferred to testing media.

### Quantitative RT-PCR Analysis

cDNA synthesis and quantitative RT-PCR analysis were performed as previously described [16].

### Western Blot Analysis

Western blot analyses were performed as previously described [25], [29], [30] with some modifications. Antibodies for Lamin (1∶1000, DSHB), GFP (1∶2000, Santa Cruze), FLAG (1∶5000, M2 antibody, Sigma) and horseradish peroxidase-conjugated anti-rabbit and anti-mouse IgG (1∶3000, Santa Cruze) were used. For detecting Dilp2-FLAG protein in the larval hemolymph, 12 µl of hemolymph was diluted in 2X sample buffer and loaded in each lane.

### Generation of Antiserum and Immunohistochemistry

dAdipoR and Dilp2 antisera were generated by the custom antibody production services from Youngin Frontier Inc. (Seoul, Korea). These antibodies were produced by the immunization of rabbits with synthetic peptides (for anti-dAdipoR, EQAEEFVRKVWEASWK & SLWDKFSEPALRPLR; for anti-Dilp2, SEKLNEVLSMVC & TRQRQGIVERC). Animal care and all experiments in Youngin Frontier Inc. were conducted with the approval of Institutional Animal Care and Use Committee (IACUC) of Youngin Frontier Inc. The animal handling protocol was in accordance with institutional and international guidelines. Anti-dFOXO was gifts from O. Puig (Merck research laboratories). Immunostaining was performed as previously described [16]. Fluorescence images were acquired using a FluoView confocal microscope (Olympus) and an AxioVert 200 M microscope with Apotome (Carl Zeiss). Fluorescence intensity for Dilp2 immunostaining and secGFP was measured as previously described [25] with some modifications. Confocal Z stacks of IPCs (1 µm step size) were obtained with identical laser power and scanning parameters. Using Image J, Z-projected images were generated with the Sum Slices projection type. The raw integrated density was measured encompassing the IPC region in each image. A group of seven IPCs in each brain hemisphere was measured separately, and the measured fluorescence intensity was normalized to the mean fluorescence of starved or brain-only cultured IPCs of *Dilp2-Gal4.*

### *Ex vivo* Culture

The brains were dissected from the larvae starved on the water for 20 h. The dissected brains were cultured in 20 µl of Schneider’s medium with or without human globular adiponectin at the room temperature for 12 h and fixed for Dilp2 immunostaining. The recombinant human globular adiponectin was purchased from R&D Systems.

### Statistical Analysis

Each experiment was repeated at least three times, and the data were presented as the mean and error bar (±S.E.M.). Student’s t-test was used for the statistical analyses and *p*<0.05 was accepted as statistically significant.

## Results

### dAdipoR, an Ortholog of the Mammalian Adiponectin Receptor 1, is Expressed in insulin Producing Cells

**Figure 1:**
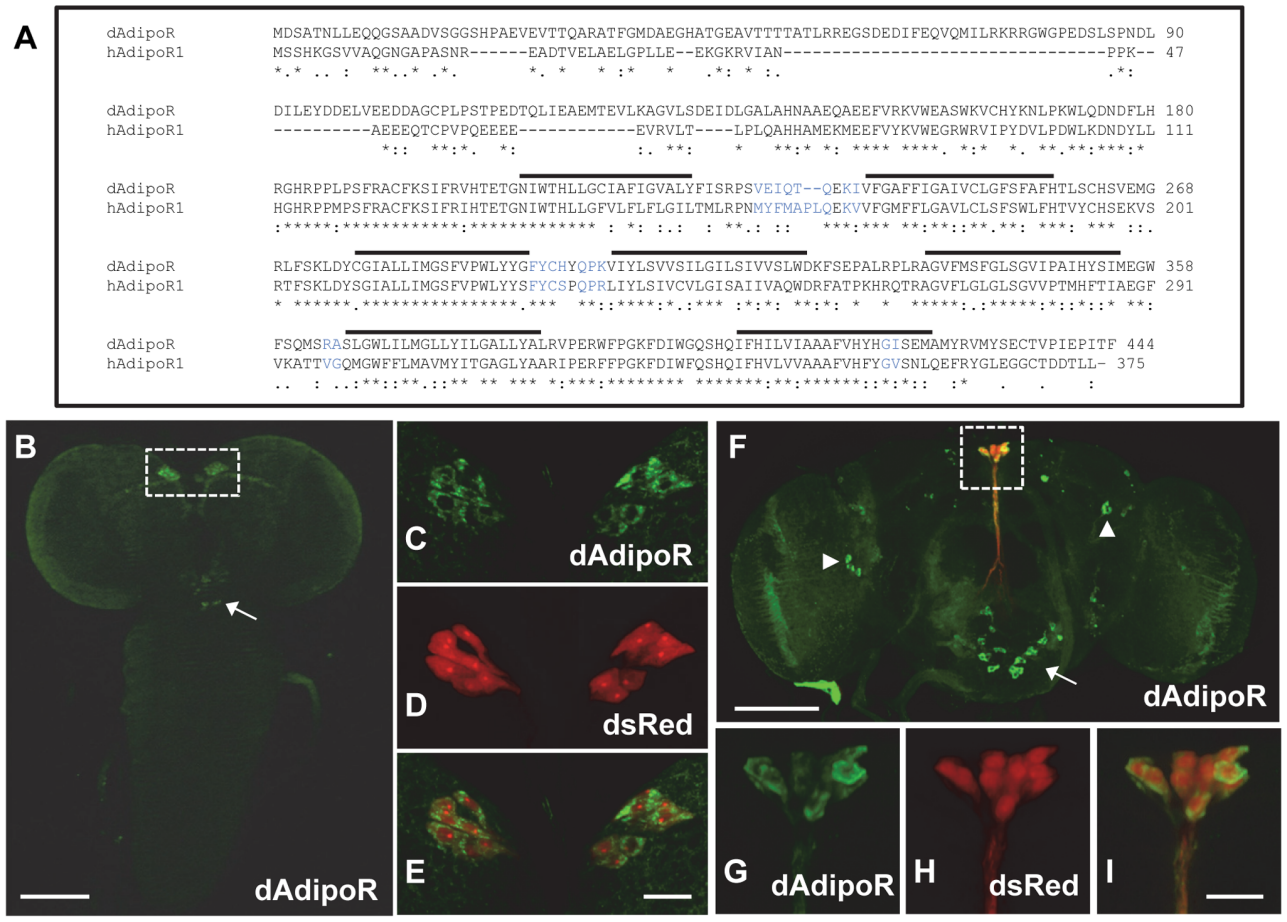
The amino acid sequence and IPC expression of *dAdipoR*. (A) Amino acid sequence comparison between dAdipoR and human adiponectin receptor 1. Seven transmembrane domain regions are marked by upper lines. The putative interaction residues with the adiponectin are marked in blue. (B-I) *Drosophila* brains from the larva (B-E) and the adult (F-I) were immunostained with the dAdipoR antibody (green) and the IPCs marker, *Dilp2>DsRed* (red). dAdipoR staining was detected in IPCs (dot boxes), SOG neurons (arrows), and lateral neurons (arrowheads). Larval IPCs in B was enlarged in C-E and adult IPCs in F was enlarged in G-I, showing that dAdipoR staining in IPCs was overlapped with the IPCs marker. Scale bars are 100 µm (B, F) and 20 µm (E, I).

To identify *Drosophila* adipokine signaling, we searched orthologous genes of mammalian adipokines and their receptors in the *Drosophila* genome. Only dAdipoR (CG5315) was found with obvious homology. dAdipoR showed 66% amino acid sequences similarity to the human AdipoR1 (Figure 1A), and hydropathy analysis predicted that dAdipoR has seven transmembrane domains (Figure S1A). According to Flybase database, there are four isoforms of *dAdipoR* transcripts, which are *dAdipoR A, B, C,* and *D*. The isoforms *A*, *C*, and *D* are translated into the same 444 amino acids protein using the same start codon located in the 2^nd^ exon of the gene, while the isoform *B* is translated into the 362 amino acids protein using the start codon located in 4^th^ exon (Figure S1B). Quantitative RT-PCR analysis revealed that the isoforms A, *C*, and *D* are predominant transcripts compared to the isoform *B* (Figure S1C). *dAdipoR* mRNA was expressed throughout all developmental stages from embryo to adult and detected in the central nervous system (CNS), imaginal disc, salivary gland, fat body, gut, and malphigian tubules of the third instar larvae (Figure S1D, E). In the *Drosophila* brain, the immunohistochemical analysis with the dAdipoR antibody revealed that dAdipoR was expressed in IPCs of larval and adult brains (Figure 1B, F, boxes). IPCs expression of dAdipoR was confirmed by the co-expression of IPCs marker, which is a DsRed reporter driven by *Dilp2-Gal4* (*Dilp2>DsRed*) in the 3^rd^ instar larval and adult brains (Figure 1C–E, G–I). Beside IPCs expression, dAdipoR expression was additionally detected in neurons of the subesophageal region of larval and adult brains (Figure 1B, F, arrows) and in lateral neurons of the adult brain (Figure 1F, arrowheads).

### *dAdipoR* Inhibition in insulin Producing Cells shows Metabolic Phenotypes

**Figure 2:**
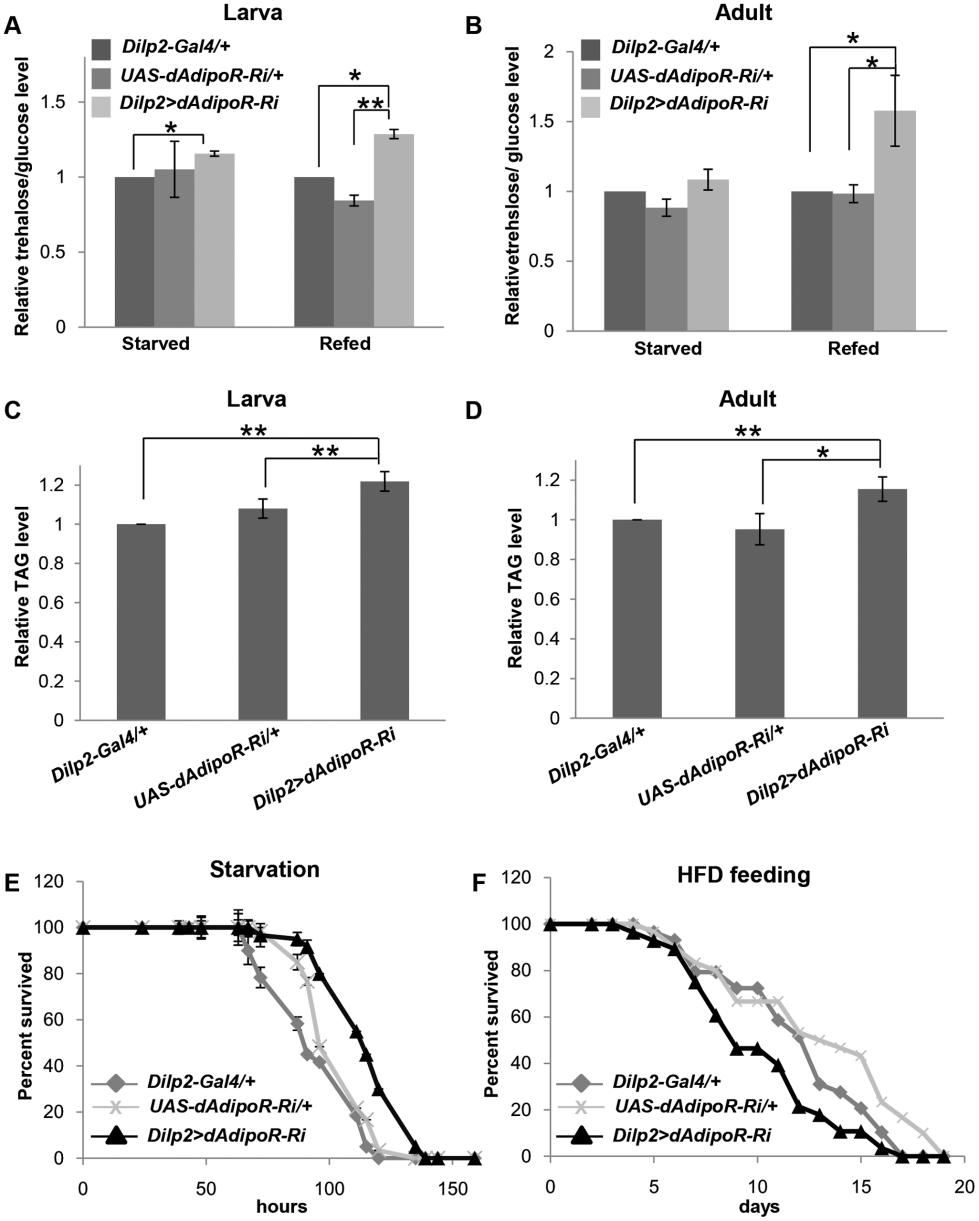
Metabolic defects of *dAdipoR* inhibition in IPCs. *Dilp2>dAdipoR-Ri* flies were increased in the hemolymph glucose levels (A, B), TAG level (C,D) of larvae and adults compared with those of *Dilp2-Gal4* and *UAS-dAdipoR-Ri* control flies. (E) *Dilp2>dAdipoR-Ri* flies showed the starvation resistance compared with *Dilp2-Gal4* and *UAS-dAdipoR-Ri* controls. (F) *Dilp2>dAdipoR-Ri* flies showed the reduced viability compared with the *Dilp2-Gal4 and UAS-dAdipoR-Ri* controls on the high fat diet. Data are presented as means ±SEM; *p<0.05, **p<0.01.

Based on the expression pattern of dAdipoR in the brain, we focused on the dAdipoR function in IPCs. To evaluate the function of dAdipoR in IPCs, we inhibited dAdipoR in IPCs by crossing IPCs-specific *Dilp2-Gal4* driver and *UAS-dAdipoR-RNAi* (*Dilp2>dAdipoR-Ri*). *UAS-dAdipoR-RNAi* can inhibit all isoforms of *dAdipoR* transcripts (Figure S1B). The quantitative RT-PCR analysis confirmed that the mRNA level of *dAdipoR* in the adult head of *Dilp2>dAdipoR-Ri* was reduced to 60% of the mRNA level of the *Dilp2-Gal4* control (Figure S2G). Moreover, immunostaining with the dAdipoR antibody showed that the dAdipoR protein level in the IPC of *Dilp2>dAdipoR-Ri* was reduced to 36% of the protein level of the *Dilp2-Gal4* control (Figure S2A–F). Since loss of Dilps by ablating IPCs results in small body size and metabolic defects [11], [12], we examined body size and metabolic phenotypes in *Dilp2>dAdipoR-Ri* flies. The body size and weight of 3^rd^ instar feeding larvae (106–108 h AEL) and 5 day-old male flies were not changed compared with those of *Dilp2-Gal4* and *UAS-dAdipoR-RNAi* control flies (Figure S3A–D). However, hemolymph trehalose and glucose levels of *Dilp2>dAdipoR-Ri* larvae and adults were significantly increased in the fed condition in comparison with those of controls and the starved conditions (Figures 2A, B). Triglyceride levels of *Dilp2>dAdipoR-Ri* larvae and adults also increased by 13–20% (Figure 2C, D). Since IPC-specific *dAdipoR* inhibition flies stored excess lipids, we investigated starvation resistance. In the starved condition, *Dilp2>dAdipoR-Ri* flies survived longer than the *Dilp2-Gal4* and *UAS-dAdipoR-Ri* control flies (Figure 2E). In contrast, in the high fat diet condition, *Dilp2>dAdipoR-Ri* flies were more sensitive to the high fat diet than the *Dilp2-Gal4* and *UAS-dAdipoR-Ri* controls. The median lifespan of *Dilp2>dAdipoR-Ri* flies was shorter compared to those of the control flies (Figure 2F). After a 5 day high fat diet, the TAG level in *Dilp2>dAdipoR-Ri* flies increased compared to that in the controls. This suggests that the shorter lifespan of *Dilp2>dAdipoR-Ri* may be due to increased lipotoxicity (Figure S3E). These metabolic phenotypes in *Dilp2>dAdipoR-Ri* flies are similar to those of *Dilps* inhibition by the ablation of IPCs [11], [12].

### dAdipoR Regulates Insulin Secretion in IPCs

**Figure 3:**
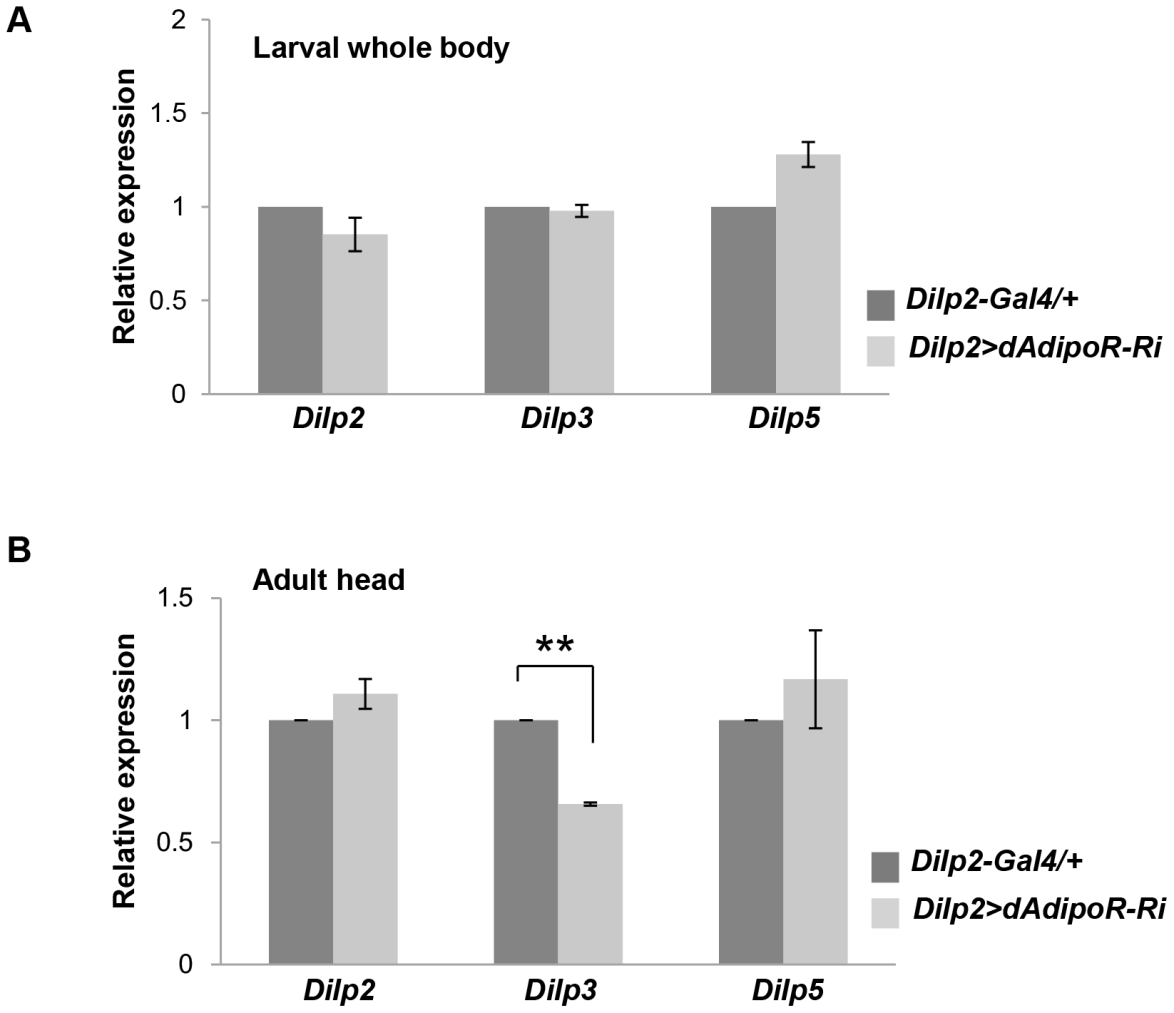
Expression of *Dilp 2, 3, 5* in *dAdipoR* knockdown flies. (A) Larval *Dilp2, 3, 5* expression levels in *Dilp2>dAdipoR-Ri* were similar to those of *Dilp2-Gal4* controls. (B) In the adult fly heads, *Dilp2* and *Dilp5* expression were similar but *Dilp3* expression was significantly reduced compared to *Dilp2-Gal4* controls (B). Data are presented as means ±SEM; **p<0.01.

**Figure 4:**
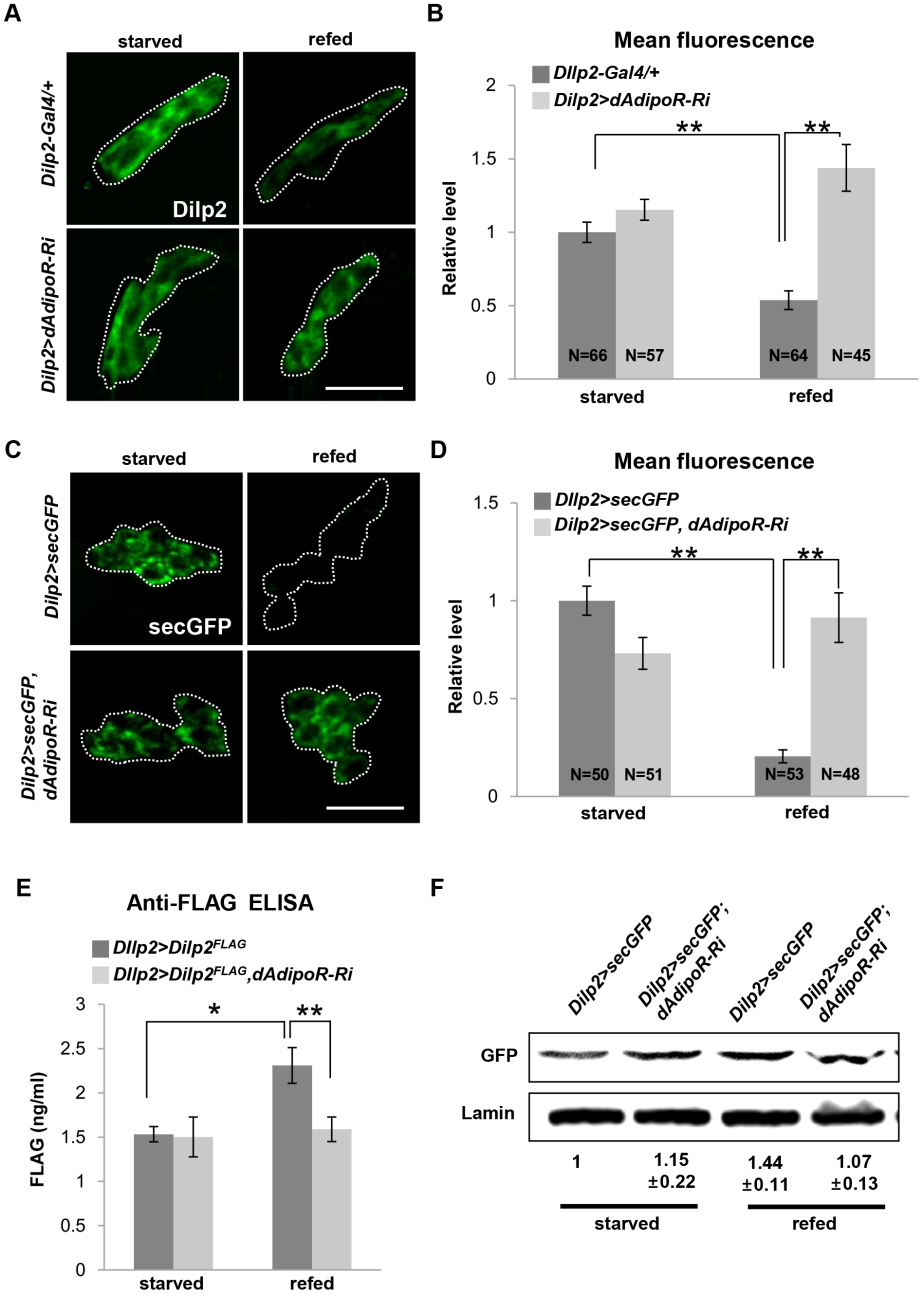
*dAdipoR* regulates Dilp secretion. (A, B) Images and relative quantifications of the Dilp2 level in larval IPCs by immunohistochemistry. In the refed condition, more Dilp2 was accumulated in IPCs of *Dilp2>dAdipoR-Ri* in comparison to those in the *Dilp2-Gal4* control. (C, D) Images and relative quantifications of the secGFP level in larval IPCs. In the refed condition, the secGFP accumulated in IPCs of *Dilp2>dAdipoR-Ri* in comparison to those in the *Dilp2-Gal4* control. (E) Anti-FLAG ELISA assay showed that the level of circulating Dilp^FLAG^ proteins was increased after refeeding in *Dilp2>Dilp^FLAG^* larvae but not in *Dilp2>Dilp^FLAG^, dAdipoR-Ri* larvae. (F) Western blot analysis showed that the secGFP level was increased after refeeding when compared to the starved condition in adult bodies of the *Dilp2>secGFP* control but not in *Dilp2> secGFP, dAdipoR-Ri* adult bodies. Data are presented as means ±SEM; *p<0.05, **p<0.01. Scale bars are 20 µm (A, C).

To identify whether metabolic phenotypes in *Dilp2>dAdipoR-Ri* flies are due to defects in *Dilps* mRNA production, we tested expression levels of *Dilp2, Dilp3* and *Dilp5* which are known to be expressed in IPCs of the fly brain. In *Dilp2>AdipoR-Ri*, mRNA expression levels of *Dilp2, Dilp3* and *Dilp5* were similar to those of the *Dilp2-Gal4* control in the larval stage, but *Dilp3* expression was slightly but significantly decreased in the adult heads relative to that of the *Dilp2-Gal4* controls (Figure 3A, B). Because the reduction of *Dilp3* expression in the adult stage does not explain the larval phenotype observed in *Dilp2>AdipoR-Ri* flies, we examined insulin secretion by the Dilp2 immunostaining in IPCs [25]. After 24 h starvation, IPCs of the *Dilp2-Gal4* larval brain were strongly stained with the Dilp2 antibody, reflecting a high accumulation of Dilp2. When the larvae were refed for 2 h, accumulated Dilp2 was secreted from IPCs and the remaining Dilp2 in IPCs was reduced to half (Figure 4A, B). However, IPCs with *dAdipoR* inhibition in the refed condition still had a high level of Dilp2 similar to the starved condition (Figure 4A, B). These data indicate that dAdipoR has a role in the secretion of Dilp2. To confirm the secretion response of IPCs, we used the secretable GFP (secGFP) as a reporter of secretion [25]. Similar to the Dilp2 staining intensity, the secGFP fluorescence intensity of *Dilp2-Gal4* IPCs diminished by 80% in the refed condition compared to that of the starved condition. However, the secGFP fluorescence intensity of *Dilp2>dAdipoR-Ri* IPCs was not reduced in the refed condition (Figure 4C, D). Because the blocking of secretion and/or the enhanced translation of Dilp2 transcripts in IPCs may have caused the increased staining of Dilp2, we assessed the Dilp2 secretion by measuring the circulating Dilp2 level in the larval hemolymph. We overexpressed FLAG-tagged Dilp2 in IPCs of the control (*Dilp2>Dilp2^FLAG^*) and the *dAdipoR* knockdown flies (*Dilp2>Dilp2^FLAG^, dAdipoR-Ri*). Then, we measured the FLAG-tagged Dilp2 protein level in the larval hemolymph with the anti-FLAG ELISA assay [27]. When the 3^rd^ instar larvae were starved for 4 h, the circulating Dilp2^FLAG^ level of the control was similar to the Dilp2^FLAG^ level of *dAdipoR* knockdown larvae (Figure 4E). The hemolymph Dilp2^FLAG^ level increased by 1.5-fold in *Dilp2>Dilp2^FLAG^* control larvae after refeeding compared to the level in the starved condition, but the hemolymph Dilp2^FLAG^ level did not change in the *Dilp2>Dilp2^FLAG^, dAdipoR-Ri* larvae after refeeding (Figure 4E). In addition, we observed that *Dilp2>Dilp2^FLAG^, dAdipoR-Ri* larvae had a lower level of hemolymph Dilp2^FLAG^ than that of *Dilp2>Dilp2^FLAG^* larvae by the Western blot analysis (Figure S4A). This result indicates that dAdipoR regulates Dilp2 secretion in larvae. To test whether dAdipoR also regulates Dilp2 secretion in adult flies, we measured secGFP in the thorax and abdomen from the *Dilp2>secGFP* control and *Dilp2>secGFP, dAdipoR-RNAi* flies using the Western blot analysis. The GFP protein level in the body of the refed *Dilp2>secGFP* control flies increased by 1.4-fold compared to that of the starved flies, whereas the GFP protein level in the body of refed *AdipoR* inhibition flies was similar to that of the starved condition (Figure 4F). These results indicate that *dAdipoR* regulates insulin secretion in IPCs of larvae and adults.

### Insulin Signaling is Reduced in the *dAdipoR Knockdown Flies*

**Figure 5:**
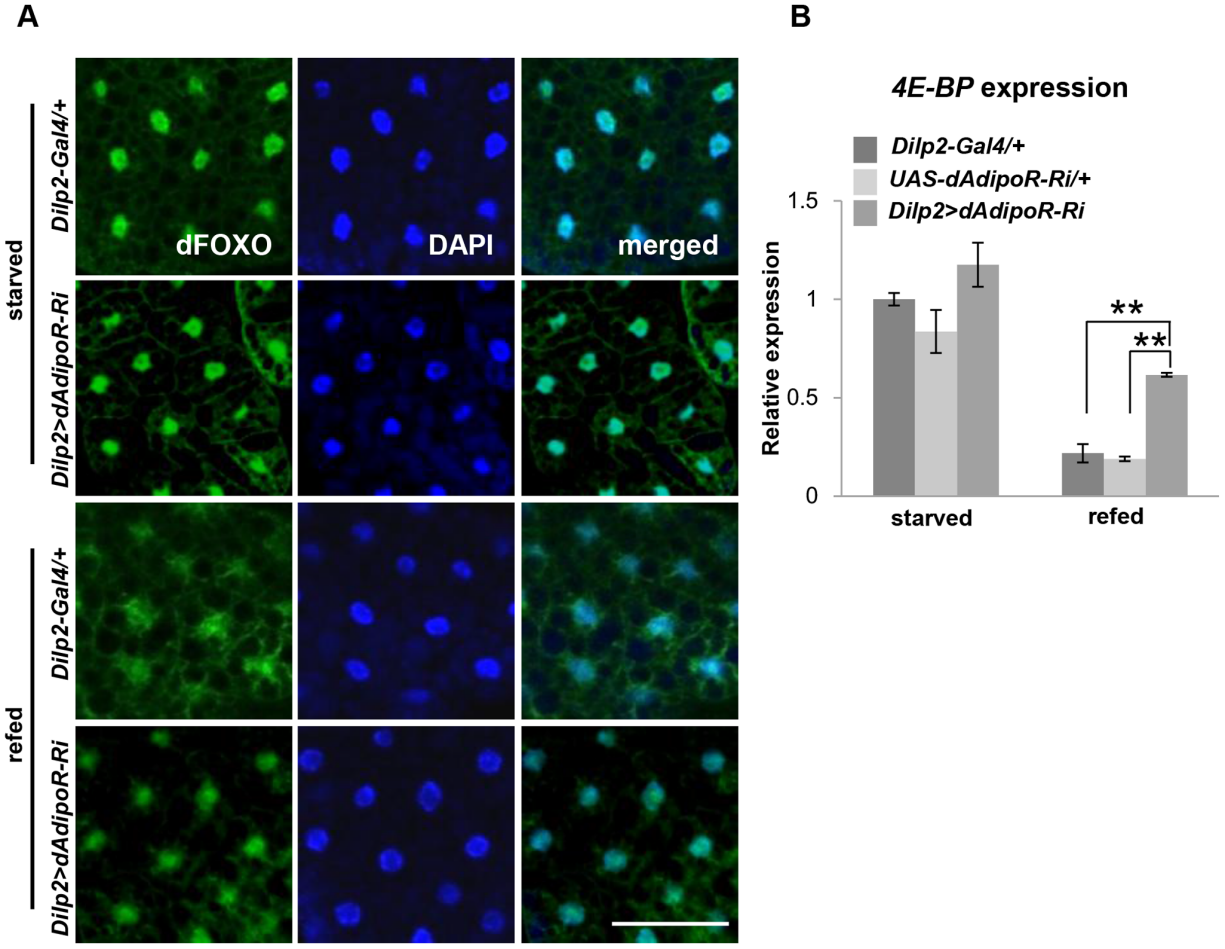
IPC-specific inhibition of *dAdipoR* reduces insulin signaling in peripheral tissues. (A) The subcellular localization of dFOXO in the larval fat body. In the starved condition, dFOXO was localized in the nuclei of the *Dilp2-Gal4* control and the *Dlip2>dAdipoR-RNAi* fat bodies. In the refed condition, dFOXO was localized in the nuclei and cytoplasm of the *Dilp2-Gal4* control fat body, but dFOXO was mainly localized in the nuclei of the *Dlip2>dAdipoR-RNAi* fat body. (B) The mRNA expression level of *4E-BP* was significantly higher in the adult body of *Dilp2>dAdipoR-Ri* than those of *Dilp2-Gal4* and *UAS-dAdipoR-Ri* controls after refeeding. Scale bar is 100 µm (A).

Since dAdipoR positively regulated insulin secretion, the *Dilp2>dAdipoR-Ri* flies would have reduced insulin signaling in insulin target tissues. To measure the activity of insulin signaling in *Dilp2>dAdipoR-Ri* flies, we examined the subcellular localization of dFOXO and the activity of dFOXO in peripheral tissues. In insects, the fat body, a homologous organ of mammalian liver and adipocytes, is the major insulin target tissue. In the starved condition, dFOXO was mainly localized in nuclei of the *Dilp2-Gal4* control and *Dilp2>dAdipoR-Ri* fat bodies. After refeeding, dFOXO was relocated to the cytoplasm in the *Dilp2-Gal4* control fat body while most dFOXO proteins were still located in the nuclei of the *Dilp2>dAdipoR-Ri* fat body (Figure 5A). Then, we measured the expression level of the dFOXO target gene *4E-BP* in adult fly bodies. In the starved condition, the expression level of *4E-BP* in *Dilp2>dAdipoR-Ri* flies were similar with those of the controls. In the refed condition, the expression level of *4E-BP* in controls decreased to 20% of the starved condition level, but the expression level of *4E-BP* in *Dilp2>dAdipoR-Ri* flies decreased to 60% of the starved condition level (Figure 5B). These data demonstrate that insulin signaling is reduced in the insulin target tissue of *Dilp2>dAdipoR-Ri* flies.

### Human Adiponectin Activated Dilp2 Secretion from the IPCs of Larval Brains

**Figure 6:**
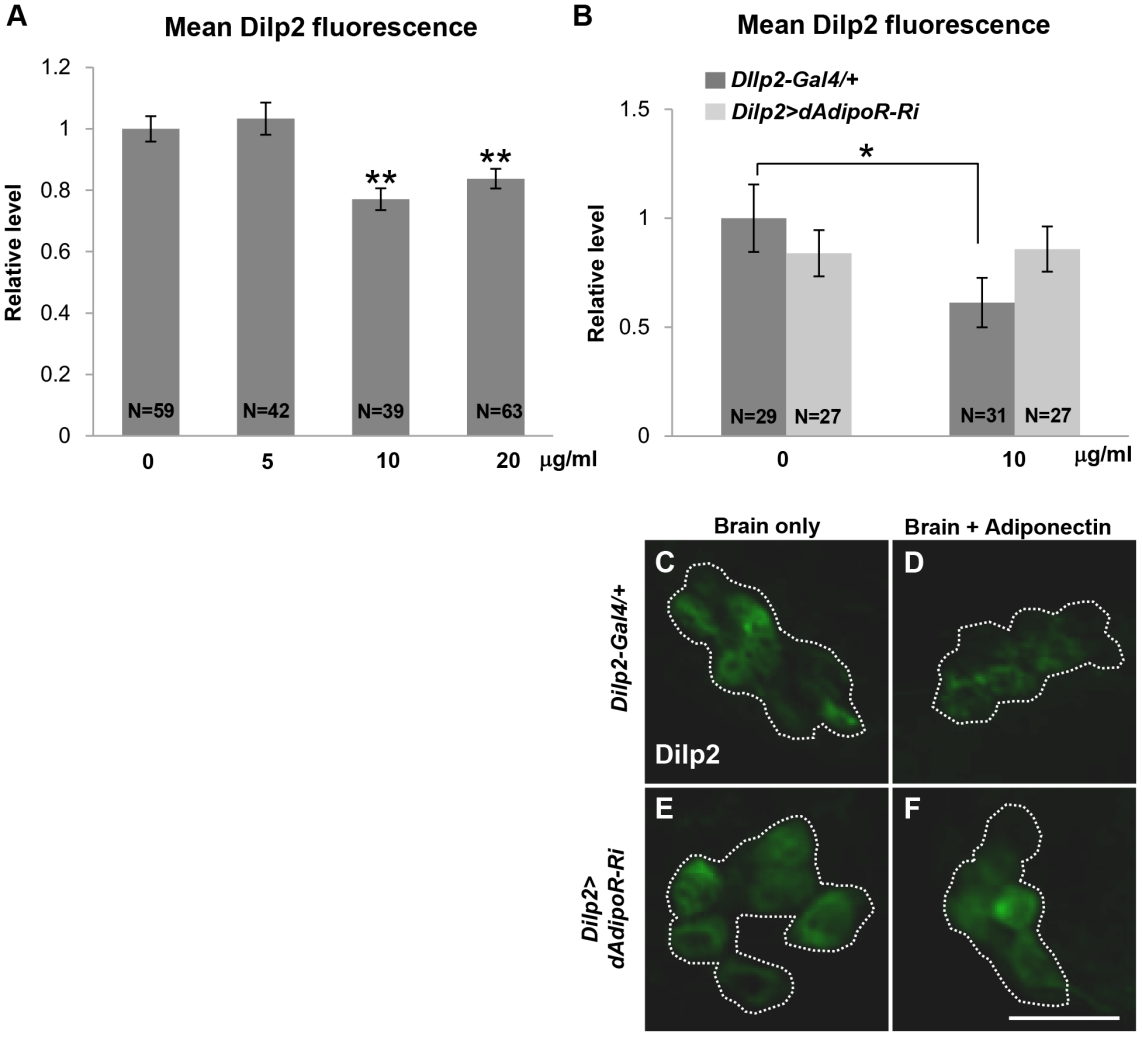
Human adiponectin induces Dilp2 secretion via *dAdipoR.* (A) The dose effect of human adiponectin on Dilp2 secretion measured by Dilp2 staining intensity. 10 to 20 µg/ml of human adiponectin significantly induced Dilp2 secretion. (B-F) Images and relative intensities of Dilp2 staining in larval IPCs after treating with human adiponectin (10 µg/ml). DIlp2 secretion induced by human adiponectin was inhibited in IPCs of *Dilp2>dAdipoR-Ri* larvae. Data are presented as means ±SEM; **p<0.01. Scale bar is 20 µm (F).

Due to the structural and functional similarities of dAdipoR with mammalian adiponectin receptors, we speculated that an adiponectin-like protein from the fat body may activate dAdipoR and induce insulin secretion. The yeast adiponectin receptor ligand osmotin can bind and activate human adiponectin receptors, suggesting that an adiponectin from one species can bind and activate adiponectin receptors in another species [31], [32]. Moreover, the amino acid residues of dAdipoR predicted to interact with an adiponectin are homologous to those of human AdipoR1 (Figure 1A) [31]. Therefore, we tested whether human globular adiponectin, which has a high binding affinity to human AdipoR1, binds to dAdipoR and induces Dilp2 secretion in larval IPCs. Three different concentrations of human adiponectin were treated to dissected *Dilp2-Gal4* larval brains (Figure 6A). 10 and 20 µg/ml of adiponectin significantly decreased Dilp2 staining intensity (23% and 16%, respectively) when compared to the untreated control, implying that human adiponectin can stimulate Dilp2 secretion (Figure 6A). Then, we examined whether inhibition of *dAdipoR* in IPCs could suppress Dilp2 secretion by the human adiponectin treatment. In *Dilp2>dAdipoR-Ri* brains, human adiponectin treatments did not change Dilp2 staining intensities in IPCs compared with non-treated controls (Figure 6B–F). This result suggests that human adiponectin binds to dAdipoR and controls Dilp2 secretion.

## Discussion

Adiponectin receptors cloned from yeast to humans play important roles in energy homeostasis across species [3], [32]–[34]. For the regulation of energy metabolism, mammalian *AdipoRs* are expressed in key metabolic organs such as hypothalamus, insulin target tissues and beta cells. Despite beta cell expression of adiponectin receptors [7], [8], their function in the beta cell is ambiguous. *In vitro* studies with islet cell lines and explanted islets report controversial roles of *AdipoRs* in the beta cells due to differences in experimental conditions [35]. Thus, beta cell specific disruption of *AdipoR*s is necessary to clarify *AdipoR* function. In this study, we found that *Drosophila* adiponectin receptor was expressed in IPCs and investigated its function by IPC-specific *dAdipoR* inhibition in flies. Inhibition of *dAdipoR* in IPCs did not affect development and viability of IPCs (data not shown) but impaired insulin secretion. Together with the secretion defect, a decrease in *Dilp3* transcript levels of adult flies was observed (Figure 3B). The reduced expression of *Dilp3* appears to have some correlation with Dilp secretion. When insulin secretion is inhibited by the overexpression of mammalian *UCP* genes in IPCs, *Dilp3* expression is decreased but expression levels of *Dilp2* and *Dilp5* are not changed [36]. The relationship between *Dilp3* expression and Dilp secretion needs further analysis. Our *in vivo* study for dAdipoR function suggests that mammalian adiponectin receptors in the beta cell may have similar roles in insulin secretion and production.

Unlike mammalian insulin, *Drosophila* insulin-like peptides regulate larval growth and energy metabolism [11], [15], [37]. In this study, however, the interference of Dilp secretion by dAdipoR reduction did not change the body size, although it clearly affected energy homeostasis. Partial reduction of the *Dilp2* mRNA level changes glucose metabolism and starvation resistance but not growth retardation partly due to the compensatory mechanism among *Dilp* genes [26]. Therefore, partial inhibition of Dilp secretion by dAdipoR reduction in IPCs may not enough for blocking growth or the compensatory mechanism.

Human globular adiponectin was able to induce Dilp secretion through dAdipoR in the *ex vivo* culture suggesting dAdipoR is a functional homologue of human adiponectin receptors and globular adiponectin like molecule would be the ligand of dAdipoR. We could not find *Drosophila* adiponectin using the amino acid sequence homology search, possibly because the *Drosophila* adiponectin-like molecule may share only structural similarities to adiponectins of other organisms. Human adiponectin and tobacco osmotin do not share the amino acid sequence homology, but they have an overlapped beta barrel structure and tobacco osmotin that can activate human AdipoR1 [31], [32]. Therefore, searching beta barrel structure proteins homologous to human globular adiponectin is one possible approach to uncover the identity of the *Drosophila* adiponectin-like molecule.

*Drosophila* adipokine signaling is not well understood yet. The fat body, *Drosophila* adipose tissue, is a source for the production of adipokines. Depending on nutrient availability, the fat body secretes humoral signals to remotely regulate IPC function [25], [38]–[40]. The amino acid signaling in the fat body releases humoral signals [25]. Recently, cytokine Upd2 was identified as a fat body humoral factor for Dilp secretion in the brain [24]. The expression of *upd2* in the fat body is regulated by sugar and lipid not amino acid [24]. These previous studies indicate that the fat body secretes multiple factors to modulate IPC function. Our findings suggest that the unidentified *Drosophila* adiponectin could be one of the fat body signals to control insulin secretion in IPCs through dAdipoR. These findings can provide an insight for the function of mammalian adiponectin receptor in pancreatic beta cells, which could be useful for therapeutic application.

## Supporting Information

Figure S1 **Domain prediction and expression of** ***dAdipoR*** **.** (A) A hydropathy plot predicted seven transmemebrane domains in the dAdipoR protein. (B) A schematic diagram of the genomic region of the *dAdipoR* gene and *dAdipoR* isoforms. The dAdipoR-RNAi targeting region and isoform specific primers are indicated by arrows. (C) Quantitative RT-PCR analysis showed expression levels of *dAdipoR* isoforms in third instar larvae of *w-*. Isofrom, *A*, *C*, *D* are major forms. (D, E) *dAdipoR* expression in all developmental stages (D) and various larval tissues (E). The expression of the *ribosomal protein 49* (*rp49*) gene was used as an internal control for the semi-quantitative RT –PCR analysis.
(TIF)

Figure S2 **Expression of** ***dAdipoR*** **in** ***Dilp2>dAdipoR-Ri*** **flies.** (A-F) Adult brain staining with the dAdipoR antibody showed IPC-specific knockdown of dAdipoR and the antibody specificity. (A, D) dAdipoR immunostaining was found in the IPCs (dot boxes) and neurons in SOG region (arrows) of the adult brain of the *Dilp2-Gal4* control and *Dilp2>dAdipoR-Ri* flies. Images of *Dilp2-Gal4* and *Dilp2>dAdipoR-Ri* brains were taken with the identical confocal setting. Intensities of dAdipoR immunostaining in neurons of the SOG region of *Dilp2-Gal4* and *Dilp2>dAdipoR-Ri* flies (C, F) are similar each other, but the intensity of dAdipoR immunostaining in *Dilp2-Gal4* IPCs (B) was stronger than that of *Dilp2>dAdipoR-Ri* IPCs (E). Scale bars are 100 µm (A) and 40 µm (B, C). (G) *dAdipoR-RNAi* in IPCs reduced the mRNA level of *dAdipoR* in adult heads. Quantitative RT-PCR performed with the primer set ABCD (Figure S1B) to detect all isoforms of *dAdipoR.*
(TIF)

Figure S3 **Normal growth and the accumulation of TAG in** ***Dilp2>dAdipoR-Ri*** **flies.** *Dilp2>dAdipoR-Ri* larvae showed similar larval weight (A) and length (B) to *Dilp2-Gal4* and *UAS-dAdipoR-RNAi* controls. The adult body weight (C) and wing size (D) of the *dAdipoR* knockdown flies were also similar to those of *Dilp2-Gal4* and *UAS-dAdipoR-RNAi* controls. (E) The high fat diet induced the accumulation of TAG in *Dilp2>dAdipoR-Ri* adult flies relative to *Dilp2-Gal4* and *UAS-dAdipoR-RNAi* controls. (F) The high level of TAG in *Dilp2>dAdipoR-Ri* larvae was rescued by the *Dilp2* overexpression in IPCs.
(TIF)

Figure S4 **Dilp2-FLAG levels in the larval hemolymph.** (A) The Western blot analysis showed that *Dilp2>dAdipoR-Ri* larvae had a lower level of circulating Dilp2-FLAG compared to *Dilp2-Gal4* control. The same amount of hemolymph (12 µl) was loaded in each lane.
(TIF)
