# Supplementary figures and images for: Drosophila Adiponectin Receptor in Insulin Producing Cells Regulates Glucose and Lipid Metabolism by Controlling Insulin Secretion
Source: PLoS One. 2013 Jul 12;8(7):e68641. doi: 10.1371/journal.pone.0068641 (PMC3709998; doi:10.1371/journal.pone.0068641)

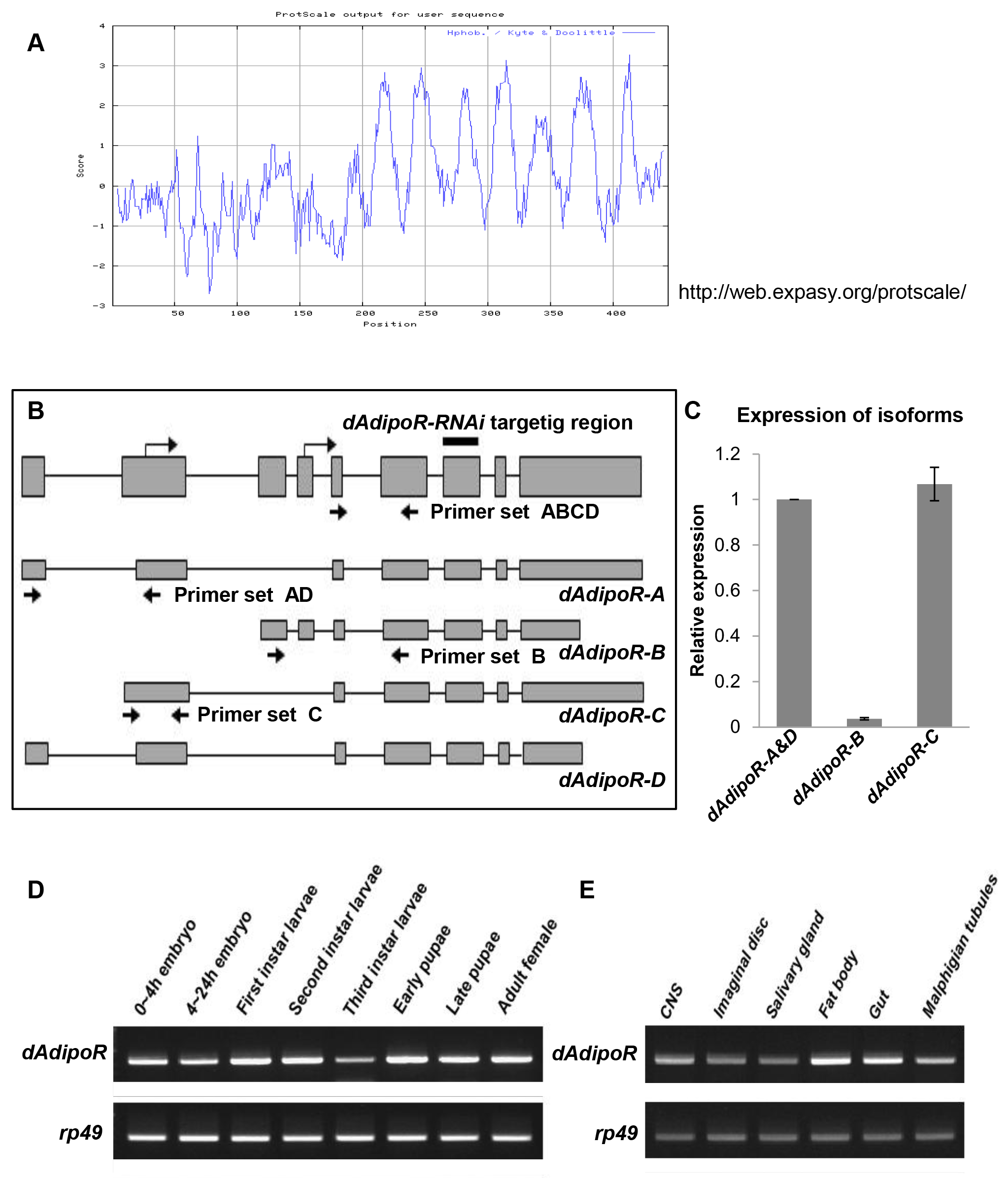

Supplement: Figure S1 — Domain prediction and expression of dAdipoR . (A) A hydropathy plot predicted seven transmemebrane domains in the dAdipoR protein. (B) A schematic diagram of the genomic region of the dAdipoR gene and dAdipoR isoforms. The dAdipoR-RNAi targeting region and isoform specific primers are indicated by arrows. (C) Quantitative RT-PCR analysis showed expression levels of dAdipoR isoforms in third instar larvae of w-. Isofrom, A, C, D are major forms. (D, E) dAdipoR expression in all developmental stages (D) and various larval tissues (E). The expression of the ribosomal protein 49 (rp49) gene was used as an internal control for the semi-quantitative RT –PCR analysis. (TIF) [file pone.0068641.s001.tif]

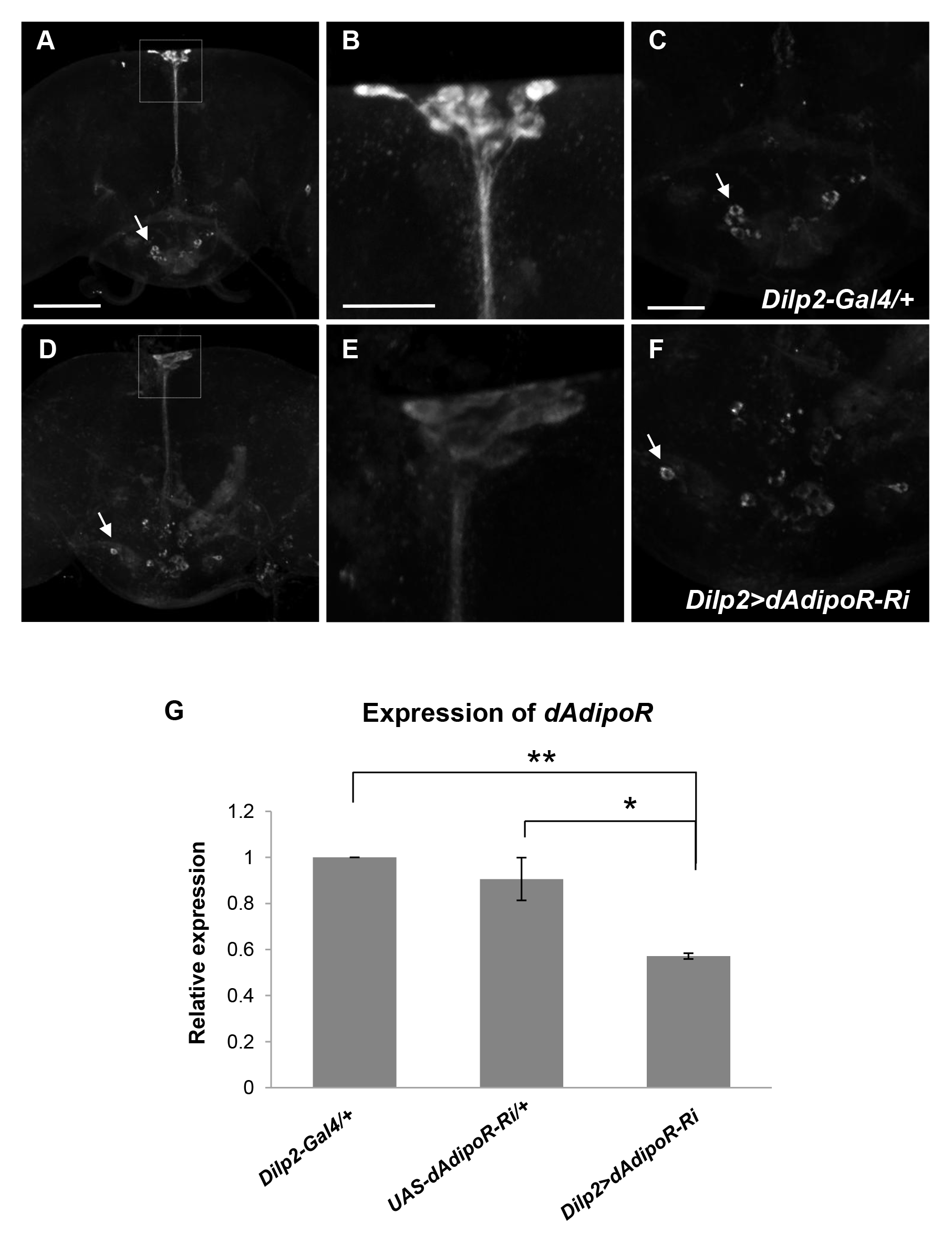

Supplement: Figure S2 — Expression of dAdipoR in Dilp2>dAdipoR-Ri flies. (A-F) Adult brain staining with the dAdipoR antibody showed IPC-specific knockdown of dAdipoR and the antibody specificity. (A, D) dAdipoR immunostaining was found in the IPCs (dot boxes) and neurons in SOG region (arrows) of the adult brain of the Dilp2-Gal4 control and Dilp2>dAdipoR-Ri flies. Images of Dilp2-Gal4 and Dilp2>dAdipoR-Ri brains were taken with the identical confocal setting. Intensities of dAdipoR immunostaining in neurons of the SOG region of Dilp2-Gal4 and Dilp2>dAdipoR-Ri flies (C, F) are similar each other, but the intensity of dAdipoR immunostaining in Dilp2-Gal4 IPCs (B) was stronger than that of Dilp2>dAdipoR-Ri IPCs (E). Scale bars are 100 µm (A) and 40 µm (B, C). (G) dAdipoR-RNAi in IPCs reduced the mRNA level of dAdipoR in adult heads. Quantitative RT-PCR performed with the primer set ABCD (Figure S1B) to detect all isoforms of dAdipoR. (TIF) [file pone.0068641.s002.tif]

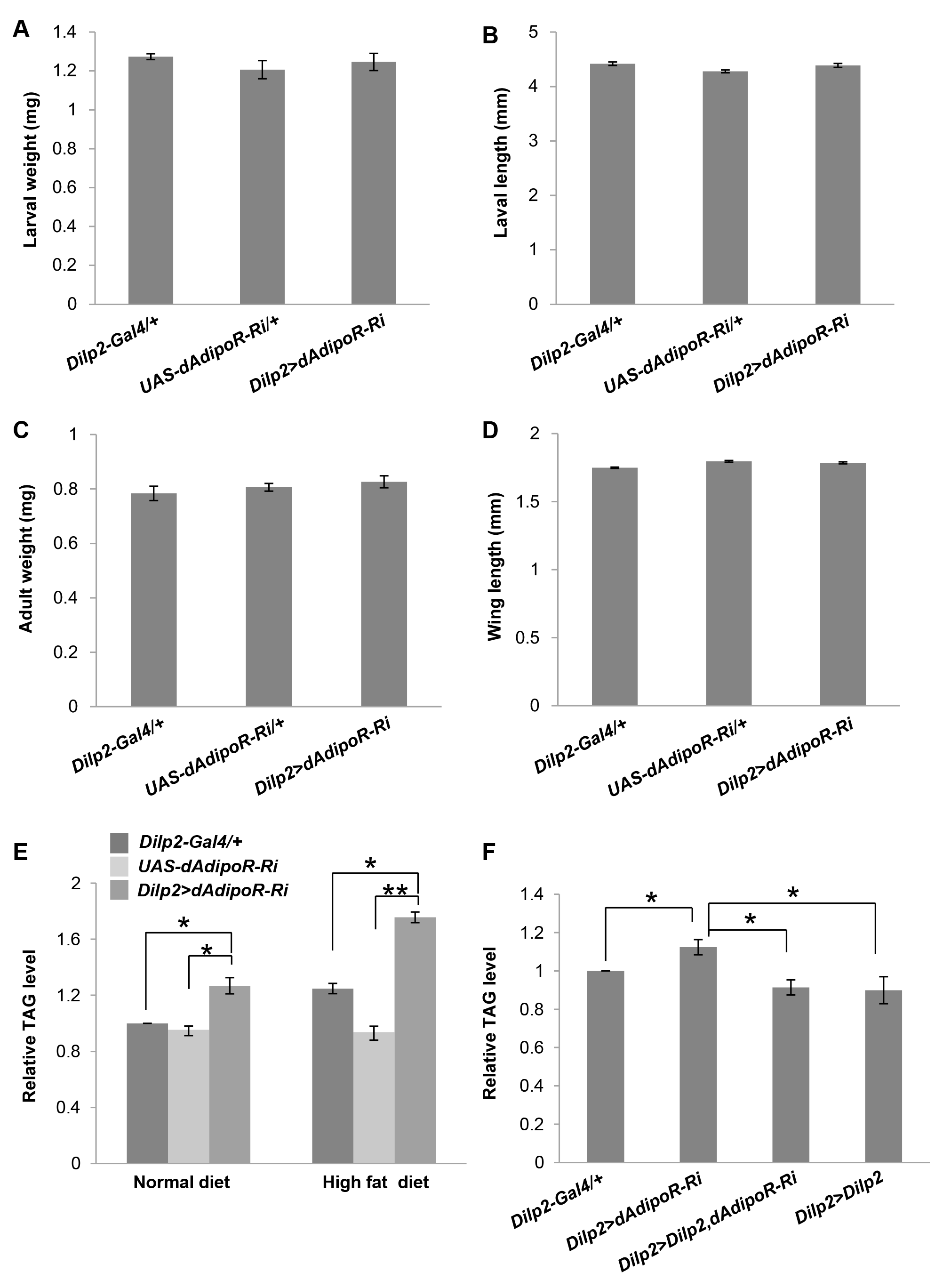

Supplement: Figure S3 — Normal growth and the accumulation of TAG in Dilp2>dAdipoR-Ri flies. Dilp2>dAdipoR-Ri larvae showed similar larval weight (A) and length (B) to Dilp2-Gal4 and UAS-dAdipoR-RNAi controls. The adult body weight (C) and wing size (D) of the dAdipoR knockdown flies were also similar to those of Dilp2-Gal4 and UAS-dAdipoR-RNAi controls. (E) The high fat diet induced the accumulation of TAG in Dilp2>dAdipoR-Ri adult flies relative to Dilp2-Gal4 and UAS-dAdipoR-RNAi controls. (F) The high level of TAG in Dilp2>dAdipoR-Ri larvae was rescued by the Dilp2 overexpression in IPCs. (TIF) [file pone.0068641.s003.tif]

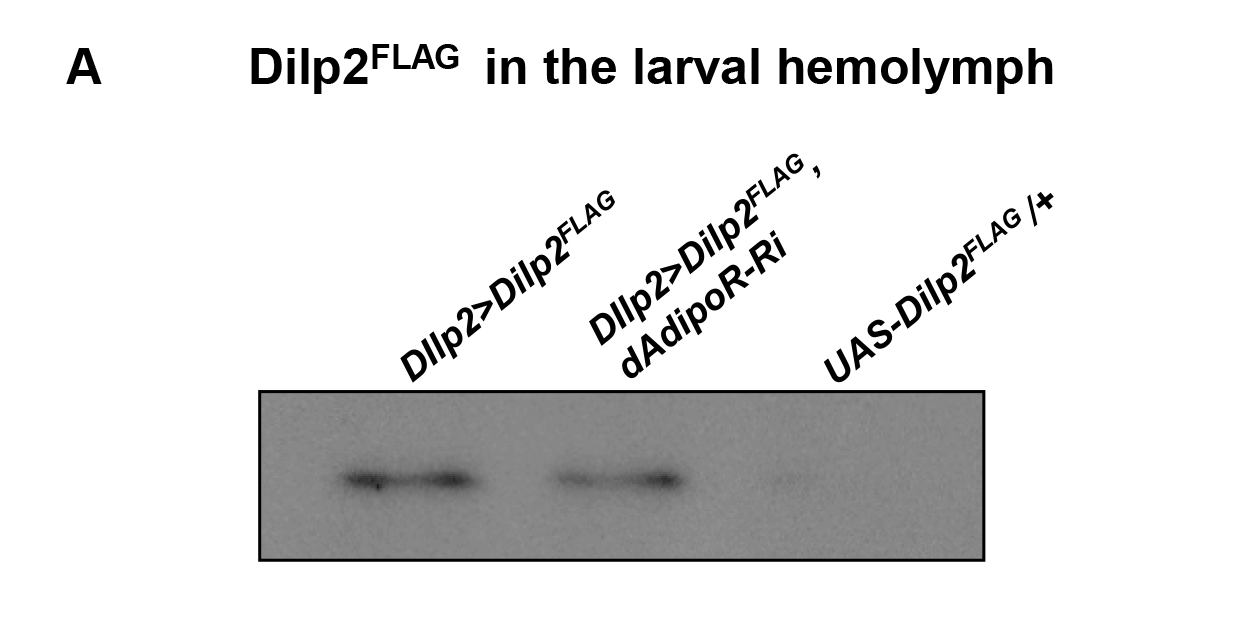

Supplement: Figure S4 — Dilp2-FLAG levels in the larval hemolymph. (A) The Western blot analysis showed that Dilp2>dAdipoR-Ri larvae had a lower level of circulating Dilp2-FLAG compared to Dilp2-Gal4 control. The same amount of hemolymph (12 µl) was loaded in each lane. (TIF) [file pone.0068641.s004.tif]
